# Calcium Hydroxylapatite-Based Fillers in Facial Rejuvenation: A Prospective, Single-Center, Unblinded Comparative Outcome Study of Radiesse^®^ vs. Rennova^®^ Diamond Intense

**DOI:** 10.3390/jcm14124072

**Published:** 2025-06-09

**Authors:** Bruna S. F. Bravo, Leonardo G. Bravo, Bárbara F. Gouvea, Marina R. B. Neves, Camila S. Nobre, Camila d. S. Silva, Carolina Machado Ozório Lopes do Nascimento

**Affiliations:** 1 Department of Dermatology, Bravo Private Clinic, Rio de Janeiro 22440-032, Brazil; 2 Innovapharma Brasil Farmaceutica Ltda, São Paulo 04578-910, Brazil

**Keywords:** calcium hydroxylapatite, dermal fillers, facial rejuvenation, biostimulation, radiesse, rennova

## Abstract

**Background/Objectives**: Calcium hydroxylapatite (CaHA)-based dermal fillers are widely used in esthetic medicine due to their dual volumizing and biostimulatory properties. Despite their rising popularity, comparative clinical outcome data evaluating different CaHA fillers remain limited. This prospective, single-center, unblinded study aimed to establish the effectiveness of the CaHA filler Rennova^®^ Diamond Intense via comparison to the well-established CaHA filler Radiesse^®^ in terms of clinical performance, safety, and patient satisfaction. **Methods**: Thirty patients (28 female, 2 male) underwent a single-session bilateral injection of Rennova^®^ Diamond Intense (right side) and Radiesse^®^ (left side) in the lower and medial posterior facial regions. Outcomes evaluated at multiple time points included dermal thickness, skin elasticity, transepidermal water loss, patient-reported outcomes (S-GAIS), physician-assessed outcomes (P-GAIS), and adverse events. **Results**: Both fillers showed improvements across all parameters. Patient-reported S-GAIS indicated predominantly “improved” outcomes at days 60 and 120, whereas physician assessments (P-GAIS) predominantly indicated “very improved” results at day 120. Ultrasound revealed increases in dermal thickness for both fillers. Similarly, improvements in skin elasticity and decreases in transepidermal water loss were observed bilaterally. Mild, transient adverse events (pain, swelling, redness, bruising) resolved spontaneously within 30 days post injection. **Conclusions**: Rennova^®^ Diamond Intense effectively increases dermal thickness, improves skin elasticity, and reduces transepidermal water loss, achieving high patient and physician satisfaction. These findings underscore its safety, versatility, and efficacy for esthetic facial rejuvenation, warranting further long-term evaluation.

## 1. Introduction

In 2023, a total of 25.4 million cosmetic minimally invasive procedures have been performed in the United States alone—an increase of more than 7% compared to the previous year [1]. Over the past decade, the field of esthetic medicine has seen substantial growth in the popularity of minimally invasive procedures designed to deliver natural-looking, lasting results with minimal downtime. Among these techniques, dermal fillers have become an essential component of facial rejuvenation strategies, offering effective treatment options for volume loss, wrinkles, and facial contour enhancement [2,3,4,5]. While conventional hyaluronic acid-based fillers primarily function through direct volumization and hydration, calcium hydroxylapatite (CaHA) fillers have introduced a unique dimension to facial rejuvenation through their dual action—immediate volumizing effects combined with long-term collagen biostimulation [6,7].

CaHA is a biocompatible, biodegradable material that has long been utilized in various medical applications, including bone regeneration and dental implants, due to its excellent safety profile and tissue compatibility [8]. In esthetic practice, CaHA fillers consist of synthetic microspheres suspended in an aqueous gel carrier, providing immediate structural support while simultaneously stimulating collagen production over time [9]. This collagen-stimulating property not only enhances the immediate esthetic outcome but also supports sustained improvements in skin elasticity, texture, and volume [10,11].

Despite their widespread use, comparative outcome data on the safety, efficacy, and patient satisfaction of different CaHA-based fillers remain limited. Existing research has predominantly involved retrospective analyses, which inherently carry potential biases and limitations. Addressing this research gap through robust, prospective studies is essential for clinicians to make informed decisions tailored to patient expectations and treatment goals.

In this prospective, single-center, unblinded study, we compared two CaHA dermal fillers, namely the novel Rennova^®^ Diamond Intense and the well-established Radiesse^®^. Specifically, we herein systematically analyzed and compared esthetic outcomes, dermal thickness, skin elasticity, trans-epidermal water loss (TEWL), and adverse effects post treatment. In addition, both patient- and physician-reported satisfaction scales were evaluated to comprehensively measure perceived and objective treatment success. The findings of this study aim to provide critical insights to practitioners regarding the performance and efficacy of Rennova^®^ Diamond Intense as a novel esthetic alternative, ultimately enhancing patient satisfaction and procedural outcomes in esthetic practice.

## 2. Materials and Methods

### 2.1. Study Setup

This prospective, single-center, and unblinded study evaluated the performance of two CaHA-based dermal fillers, Rennova^®^ Diamond Intense and Radiesse^®^, each administered in a single-session treatment of the lower medial and posterior facial region. Conducted from August through December 2024 in the senior author’s private practice, the investigation was considered low-risk, involving standard clinical procedures rather than experimental interventions. All participants provided informed consent. This study adhered to the principles outlined in the 1996 Declaration of Helsinki and complied with regional regulations and good clinical practice guidelines. Ethical approval for this study was granted by the Hospital Pró-Cardíaco Ethics Committee under approval number 7.466.526 and CAAE code 86343325.9.0000.5533.

### 2.2. Inclusion and Exclusion Criteria

We applied strict inclusion and exclusion criteria to ensure patient safety, as well as study integrity and validity. Exclusion criteria were explicitly defined to eliminate any potential confounding factors. Individuals under 18 were excluded to maintain an adult sample. Pregnant or breastfeeding women were not enrolled, due to the inherent risks to both the mother and child. Participants with a history of autoimmune diseases were not eligible, to avoid complications related to immune responses, while those using immunosuppressants or anti-inflammatory medications were also excluded to prevent any interference with treatment outcomes. Additionally, individuals with permanent dermal fillers—such as polymethyl methacrylate (PMMA) or silicone—were not considered, given the possibility of altered treatment effects or adverse reactions. Candidates who had received temporary or semi-permanent cortical fillers within the past 12 months were also ineligible, ensuring that recent cosmetic procedures did not bias the findings. Furthermore, patients using topical or oral products that could significantly affect their skin condition were excluded. To further minimize confounding variables, patients were required to observe a washout period of at least 12 months prior to enrollment for any other esthetic facial treatments, including injectables, energy-based devices, or surgical interventions. This restriction was maintained throughout the study period.

Eligible participants for this study were adults aged 30 to 75 years with visible signs of mild to moderate facial aging, including volume loss, reduced skin elasticity, or fine to moderate rhytids in the lower medial and posterior facial regions. Participants were required to have no known allergies or hypersensitivity to any components of the dermal fillers or local anesthetics used. Additionally, participants had to be willing and able to comply with all study-related procedures, including scheduled follow-up visits and completion of post-treatment diaries. Further details are presented in Table 1. All subjects provided written informed consent after receiving a thorough explanation of this study’s purpose, methodology, potential risks, and expected benefits (Table 1). 

### 2.3. Product Specification

Rennova^®^ Diamond Intense is a biodegradable, viscous, non-pyrogenic, semi-solid, opaque, injectable, and sterile dermal implant consisting of synthetic calcium hydroxylapatite microspheres which arrives suspended in an aqueous gel vehicle composed of glycerol, carboxymethylcellulose, and phosphate buffer.

Radiesse^®^ is an injectable, sterile, non-pyrogenic, semi-solid, opaque filler composed primarily of synthetic calcium hydroxylapatite which arrives suspended in a gel carrier made of glycerin, sodium carboxymethylcellulose, and sterile water for injection.

### 2.4. Procedure

First, the target skin area in the medium and lower thirds of the posterior face behind the ligamental line was cleansed using a 0.5% alcoholic chlorhexidine solution, followed by the application of a 4% lidocaine-based anesthetic cream for 30 min. Once the cream was removed, injections were administered in the determined regions. The preauricular region was specifically selected due to its anatomical consistency, low mobility, and reliable soft tissue plane, which allow for a reproducible injection depth and accurate assessment of filler performance across all patients. While Rennova^®^ Diamond Intense was injected on the right facial hemiface, Radiesse^®^ was injected on the left facial hemisphere in the lower medial and posterior regions. The side allocation was not randomized but standardized across all patients to ensure procedural consistency and reduce operator variability. As both the injector and patients were aware of the side assignments, this study was conducted in an unblinded fashion.

The temporal areas served as anatomical controls without any active filler injection. Specifically, the control area was the anterior temple (upper zygomatic region), which is non-hair-bearing and not subject to the variability found in more distant facial zones. This area is routinely used in clinical research as a comparator in facial esthetic studies due to its relatively similar skin characteristics—such as thickness and texture—to the lower and middle face. The choice of the anterior temporal region allowed for a balanced, reproducible comparison while avoiding areas such as the perioral, periorbital, or frontal zones, which differ significantly in anatomy and were not treated in this study.

A linear retro-injection technique, without bolus, was employed for all treatments. Injections were performed strictly within the subcutaneous plane at three key anatomical locations: (i) the preauricular area, (ii) the mandibular angle region, and (iii) the posterior cheek. Periosteal injections were explicitly avoided to minimize the risk of vascular compromise and unnecessary invasiveness.

Filler placement depth was verified through a two-step process: (1) real-time clinical assessment, including tactile feedback and a visual confirmation of tissue displacement during injection, as is standard in subcutaneous esthetic techniques, and (2) post-injection ultrasonographic evaluation to confirm accurate filler deposition within the intended subcutaneous layer. A single experienced dermatologist performed all injections. The single injecting dermatologist had over 15 years of experience using Radiesse^®^ in clinical esthetic practice and received formal, product-specific training on Rennova^®^ Diamond Intense prior to initiating this study. Although Rennova^®^ was newly introduced to the practice specifically for this comparative evaluation, the injector’s expertise in calcium hydroxylapatite-based fillers and adherence to standardized injection protocols ensured the consistent, competent administration of both products across all patients. Immediately after the procedure, a massage with a 2% chlorhexidine degerming solution was applied to ensure even distribution of the product. Patients were advised to perform this massage for 3 min, 3 times daily for 3 days. Each injection site was covered with a sterile occlusive dressing.

### 2.5. Global Aesthetic Improvement Scale Assessment (GAIS)

Patient satisfaction was assessed using the Subjective Global Aesthetic Improvement Scale (S-GAIS) at both 60 and 120 days post treatment. This five-point scale measures the overall enhancement in appearance compared to the pre-treatment state, with scores indicating exceptional improvement (1), very improved (2), improved (3), unchanged (4), or worsened (5). Instead of evaluating separate elements, it considers the complete esthetic outcome.

The Physician Global Aesthetic Improvement Scale (P-GAIS) provides an additional, expert viewpoint on esthetic outcomes by two independent evaluators. Conducted on day 120 post injection, it follows the same five-point structure as the Subject Global Aesthetic Improvement Scale (S-GAIS). By mirroring the patient-reported assessment, the P-GAIS allows for a direct comparison between self-perceived results and clinical evaluations, thereby offering a comprehensive overview of esthetic improvement (Table 2). 

### 2.6. Patient Diary

Patients were provided with a diary to document any adverse effects they experienced during the 30 days following the procedure. They were asked to record specific symptoms or signs—such as “pain”, “swelling”, “hardening”, “redness”, “formation of lumps”, “bruising or hematoma”, and “change of color”—and to rate the severity of each as mild, moderate, or severe. These diaries were then collected and reviewed during the 30-day follow-up visit.

### 2.7. Clinical Evaluation

Each patient underwent ultrasonographic evaluation to assess changes in dermal thickness at five scheduled visits: days 1, 30, 60, 90, and 120. The DermaLab^®^ Combo-4 device (CORTEX TECHNOLOGY, Aalborg, Denmark) was utilized for precise ultrasound visualization of superficial skin structures, including the epidermis, dermis, and subcutaneous tissue. Patients remained seated at a 45° angle to minimize potential skin distortions caused by gravitational forces or movement. The transducer was gently placed on the skin surface, separated by a thick layer of Aquasonic Clear Ultrasound Gel (Parker Laboratories Inc., Fairfield, NJ, USA) to ensure optimal acoustic coupling while avoiding compression. Dermal thickness measurements were performed bilaterally at two standardized anatomical landmarks: the pretragal region and bilaterally in the temporal region. These reference points were clearly marked on the skin using a dermatological marking pencil for consistency across assessments. Ultrasound images were acquired using standardized, reproducible device settings, ensuring accuracy and comparability of dermal thickness measurements throughout this study.

Skin elasticity reflects the skin’s ability to return to its original shape after being stretched or displaced. This quality is primarily influenced by the presence and integrity of collagen and elastin fibers within the dermal layer. In this study, skin elasticity was quantitatively evaluated using the DermaLab^®^ Combo-4 device (CORTEX TECHNOLOGY, Aalborg, Denmark). At each clinical evaluation (days 1, 30, 60, 90, and 120), measurements were performed bilaterally in the pretragal region and the temporal region. Skin elasticity was assessed by measuring “retraction time”, defined as the time in milliseconds required for the skin to retract from its peak elevation (following a standardized vacuum-induced elevation) back to 33% of this peak height. Shorter retraction times indicate better elasticity, reflecting healthier, more resilient skin. This quantitative approach ensures the objective monitoring of skin elasticity improvements over the treatment period, providing valuable insights into how effectively each filler promotes tissue remodeling and structural support.

Transepidermal water loss (TEWL) is a critical indicator of the skin’s barrier function, measuring the rate at which water evaporates through the epidermis to the external environment. A lower TEWL generally indicates healthier, more intact skin, whereas higher values may suggest compromised barrier integrity or skin damage. TEWL was assessed using the DermaLab^®^ Combo-4 (CORTEX TECHNOLOGY, Aalborg, Denmark) at five clinical visits (days 1, 30, 60, 90, and 120). Evaluations occurred bilaterally at the previously established measurement sites—the pretragal and temporal regions. The device’s probe was gently positioned on the skin’s surface, accurately capturing evaporation rates in grams per square meter per hour (g/m^2^/h). This non-invasive procedure provides quantitative data on changes in skin hydration and barrier effectiveness, helping clinicians understand the impact of CaHA filler treatments on overall skin health. Monitoring TEWL throughout the study period offered insights into whether filler injections improved skin hydration status by enhancing skin barrier function, a factor closely linked to the overall health, integrity, and rejuvenation capacity of treated skin areas.

### 2.8. Statistical Analysis

Differences between follow-up periods and facial hemispheres within the investigated groups were determined using Wilcoxon signed-rank tests. Comparisons of parameters across multiple time points were analyzed using ANOVA. All calculations were performed using SPSS Statistics 27 (IBM, Armonk, NY, USA), and differences were considered statistically significant at a probability level of ≤0.05 to guide conclusions. Results are presented as median values and their respective interquartile (IQR) range, unless indicated differently.

## 3. Results

### 3.1. Demographics

The study population comprised 30 patients (28 females, 2 males) with a mean age of 49 ± 8.2 years.

### 3.2. Global Aesthetic Improvement Scale Assessment (GAIS)

S-GAIS on post-injection day 60 was rated as “exceptional improvement” in 3 (11%), “very improved” in 4 (15%), “improved” in 13 (48%), and “no change” in 7 (26%) patients. S-GAIS on post-injection day 120 was reported as “exceptional improvement” in 2 (8.7%), “very improved” in 7 (30%), “improved” in 9 (39%), and “no change” in 5 (22%) patients. The mode of S-GAIS on post-injection days 60 and 120 was 3 (“improved”).

**Figure 1:**
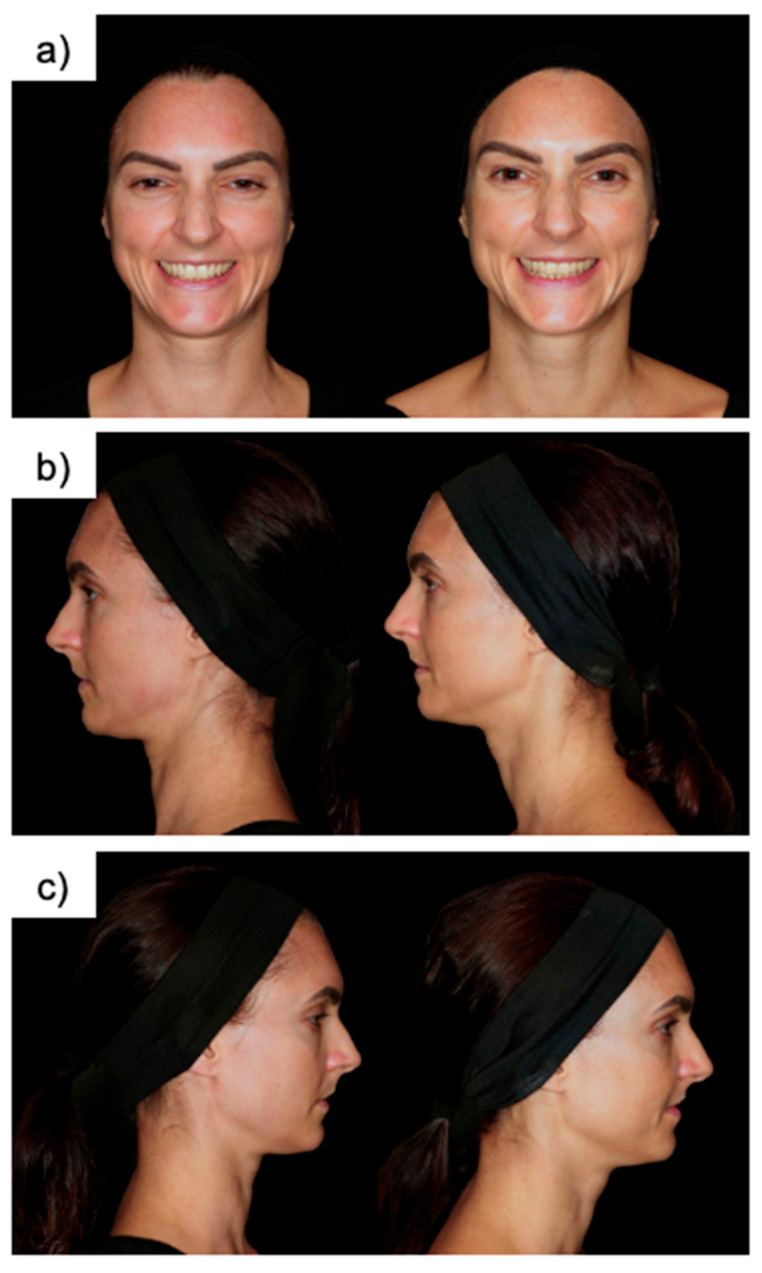
Patient photographs from different perspectives and postures before (**left**) and after (**right**) CaHA filler injection. (**a**) The frontal upright photograph, (**b**) left upright photograph, and (**c**) right upright photograph. These standardized clinical images illustrate the visible esthetic outcomes of calcium hydroxylapatite filler treatment at 120 days post injection. Improvements are most notable in the contour, volume, and firmness of the lower and posterior facial regions, particularly along the jawline and preauricular areas. All photographs were taken under identical lighting, positioning, and camera settings to ensure objective visual comparison.

P-GAIS on post-injection day 12 was rated as “exceptional improvement” in 2 (6.7%), “very improved” in 22 (73%), “improved” in 6 (20%), and “no change” in 0 (0%) patients. The mode of P-GAIS on post-injection day 120 was 2 (“very improved”) (Figure 1).

### 3.3. Patient Diary

The most common complaints raised by patients were pain (*n* = 24; 80%), followed by swelling (*n* = 15; 30%), hardening (*n* = 11; 37%), redness (*n* = 9; 30%), the formation of lumps (*n* = 8; 27%), bruising or hematoma (*n* = 7; 23%), and change of color (*n* = 4; 13%). All of these complaints resolved by postoperative day 30 at the latest.

### 3.4. Ultrasound Measurements

In the tragus region, the initial ultrasonographic assessment of skin thickness on day 1 showed 1415 µm (IQR 1248–1514) on the right and 1488 µm (IQR 1251–1620) on the left. Over the study period, the right side increased by 210 µm (IQR −3.3–358), whereas the left side increased by 66 µm (IQR −128–329). These differences in thickness change between the two sides were not statistically significant (*p* = 0.29). By day 90, thickness measurements rose to 1627 µm (IQR 1340–1702) on the right and 1575 µm (IQR 1248–1781) on the left.

**Figure 2:**
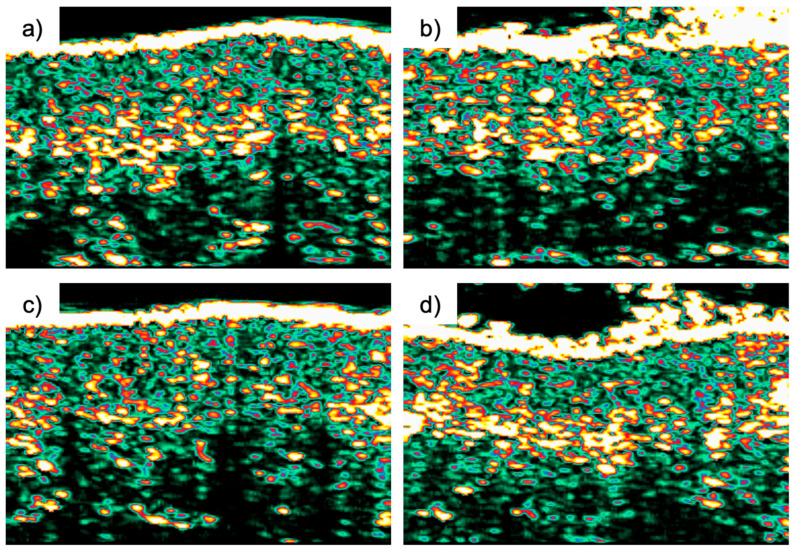
Ultrasound measurements in the tragus regions before and after filler injection assessing dermal thickness. (**a**) Dermal ultrasound before Rennova^®^ Diamond Intense injection, (**b**) dermal ultrasound after Rennova^®^ Diamond Intense injection, (**c**) dermal ultrasound before Radiesse^®^ injection, and (**d**) dermal ultrasound after Radiesse^®^ injection. The images were acquired using high-frequency ultrasonography (DermaLab^®^ Combo-4), which captures variations in skin density and echogenicity. The brighter, more reflective upper layers correspond to the dermis, while the darker areas below represent subcutaneous tissue. Increases in dermal thickness and echogenic signal after injection indicate enhanced structural density and collagen biostimulation, as shown by the more prominent and broader bright zones in panels (**b**,**d**) compared to their respective baseline images (**a**,**c**).

ANOVA confirmed that although thickness values changed over time, the differences between the two sides remained stable throughout the study period (Figure 2).

### 3.5. Skin Elasticity

In the tragus region, the initial retraction time on day 1 was 231 ms (IQR 188–422) on the right and 212 ms (IQR 160–282) on the left. Over the study period, the right side exhibited a decrease of 57 ms (IQR −212 to −32), while the left side shortened by 38 ms (IQR −81 to −7). These changes between the two sides were not statistically significant (*p* = 0.28). By day 90, retraction times decreased to 164 ms (IQR 145–192) on the right and 154 ms (IQR 141–176) on the left.

ANOVA confirmed that while retraction times changed over time, the inter-side differences remained similar throughout the study period.

### 3.6. Trans-Epidermal Water Loss (TEWL)

In the tragus region, baseline TEWL on day 1 was 7.7 g/m^2^/h (IQR 5.7–10) on the right and 5.4 g/m^2^/h (IQR 4.3–12) on the left. Over the study period, the right side decreased by 1.1 g/m^2^/h (IQR −5.6–1.2) and the left side decreased by 1.0 g/m^2^/h (IQR: −7.0–2.4), with no statistically significant difference in gain (*p* = 0.83). By day 90, TEWL declined to 6.4 g/m^2^/h (IQR 4.5–7.6) on the right, while it increased slightly to 6.0 g/m^2^/h (IQR 3.2–7.4) on the left.

ANOVA confirmed that while TEWL evolved over the time points, the inter-side differences remained stable throughout the study period.

## 4. Discussion

The increasing popularity of minimally invasive esthetic procedures underscores the need for rigorous comparative assessments of different dermal fillers. This prospective, single-center, unblinded study compared two commercially available calcium hydroxylapatite (CaHA)-based dermal fillers—Radiesse^®^ and Rennova^®^ Diamond Intense—focusing on esthetic outcomes, skin quality parameters, and patient and clinician satisfaction.

In general, both patients and physicians perceived a clear enhancement in facial appearance following CaHA injections, as reflected in S-GAIS ratings predominantly at the “improved” level and P-GAIS ratings at the “very improved” level. These findings align with Beer et al., who evaluated CaHA in 19 patients with mid-face volume loss over a six-month follow-up period, demonstrating sustained improvements in both patient- and physician-reported outcomes [12]. Similarly, Wollina et al. reported that “very improved” S-GAIS and P-GAIS ratings persisted for up to three years in individuals treated with Radiesse^®^ for mid- and lower-face rejuvenation, with subjects receiving annual maintenance injections [13]. Together, these studies substantiate the durable, positive impact that CaHA fillers can have on patient satisfaction and clinical assessments of esthetic improvement.

Our findings regarding transient, mild adverse events such as pain, swelling, redness, and bruising mirror data from previous clinical evaluations, emphasizing an overall favorable safety profile for both the Rennova^®^ Diamond Intense and Radiesse^®^ CaHA-based fillers [14]. A literature review by Kadouch, including 2779 patients who received facial CaHA injection, reported a rate of adverse events in 173 (3%) patients [7]. The assessed types of adverse events included nodules, persistent inflammation or swelling, persistent erythema, and overcorrection. Moreover, a study by Marmur et al. in 50 subjects reported significantly less pain post injection when CaHA was premixed with 2% lidocaine [15]. However, adverse events such as bruising, itching, redness, or swelling were reported in about half of the patients regardless.

While we did not find significant differences in the effect on dermal thickness, skin elasticity, and transepidermal water loss between the Rennova^®^ Diamond Intense and Radiesse^®^ CaHA filler, our findings support the established efficacy in the literature. For example, a study by Lapatina et al. enrolling 10 female patients reported significantly increased skin elasticity and dermal thickness upon subdermal injection of Radiesse^®^ [10]. These findings were corroborated by a study of Gonzáles et al. in 15 subjects who underwent punch biopsy of the right infra-auricular areas before and after injection of CaHA on day 180 [16]. They found a percentage change in elastic fibers varying between 29% and 179% at 6 months in comparison with baseline. Although not significant, they also reported a mean percentage change in proteoglycans of 76%. This is in alignment with another study by Yutskovskaya et al., who also identified an increase in skin elasticity and dermal thickness in ultrasound assessment in a cohort of 20 patients [11]. More specifically, they reported significant increases in collagen 1 and 3 expression, as well as increased staining intensity for elastin and angiogenesis markers persisting throughout the follow-up period. While evaluating the efficacy of diluted CaHA injection for dorsal hand treatment in 15 women, Figueredo et al. report significant improvements in skin viscoelasticity measures and ultrasonography for dermal parameters [17]. Furthermore, they found a significant increase in mean total collagen density post injection. With regard to transepidermal water loss, our findings are in alignment with the limited literature. While comparing hyaluronic acid-based fillers to CaHA-based fillers injected into the dorsum of the hands, Kim reported improved transepidermal water loss in both hands, indicating that the skin became healthier and had a stronger barrier upon filler injection [18]. Similar results have been reported by Lee et al. upon intradermal injection of a hyaluronic acid-based filler in 20 patients who demonstrated signs of skin aging. Transepidermal water loss, as well as skin hydration and elasticity, exhibited significant improvements over time compared with baseline measurements [19]. Based on these objective measurements of outcome, both Rennova^®^ Diamond Intense and Radiesse^®^ demonstrated comparable performance.

The findings of this study have relevant implications for clinical practice, demonstrating that Rennova^®^ Diamond Intense offers excellent esthetic outcomes, safety, and patient satisfaction to the widely established Radiesse^®^. Given the performance of Rennova^®^ Diamond Intense regarding dermal thickness, skin elasticity, and transepidermal water loss, practitioners can confidently select between these two CaHA-based fillers based on patient-specific factors such as cost, availability, and individual preferences. Furthermore, this study contributes valuable prospective data that enhance the clinical evidence supporting the interchangeable use of these fillers, ultimately promoting more personalized and informed decision making in esthetic medicine. Future research should consider longer-term evaluations and multicenter designs to further validate these findings across diverse patient populations and broader clinical contexts.

### Limitations

This study has several limitations that must be acknowledged. Although this study provides valuable preliminary insights, its conclusions are inherently limited by the small sample size of 30 patients, which may reduce the sensitivity to detect subtle subgroup differences, such as those related to age, skin type, or baseline laxity. In addition, the 4-month follow-up period captures only initial tissue responses, potentially missing the longer trajectory of collagen remodeling and late-onset adverse events. Extending follow-up to at least 12 months, ideally within a larger, stratified cohort, would provide a more comprehensive understanding of long-term efficacy, delayed complications, and the evolution of patient satisfaction as early post-treatment changes stabilize. Additionally, the single-center design of our study limits the diversity of the patient population, and larger multicenter trials are warranted to validate these findings across different clinical settings, regions, and demographics. Moreover, despite rigorous inclusion and exclusion criteria, individual variations in skin type, lifestyle factors, and compliance with post-treatment care may influence the outcomes observed, highlighting the need for larger and more heterogeneous cohorts. Furthermore, the allocation of Rennova^®^ Diamond Intense to the right and Radiesse^®^ to the left side of the face was not randomized. This fixed side assignment was chosen to maintain procedural consistency and reduce operator-related variability during bilateral injections. However, the lack of randomization introduces a potential bias and may have influenced side-specific outcomes due to anatomical asymmetry or unconscious procedural differences. Additionally, as this was an unblinded study, both the injector and participants were aware of the treatment allocation, which could have influenced subjective outcome reporting. An additional practical limitation of this study is that Rennova^®^ Diamond Intense, while demonstrating comparable effectiveness to Radiesse^®^, is not currently available in the United States, potentially limiting its immediate clinical applicability in this region. While ultrasonography provided valuable structural data, it is an operator-dependent imaging method and thus more susceptible to inter-observer variability and reduced objectivity compared to quantitative assessments. The evaluation relied primarily on two objective parameters—skin elasticity and transepidermal water loss (TEWL)—which, although standardized and reproducible, may not fully capture the range of tissue remodeling or collagen stimulation occurring at the histological level. Future studies could provide deeper mechanistic insights by incorporating histological analyses, which would allow for the direct, tissue-level evaluation of collagen remodeling and extracellular matrix changes. Finally, this study was conducted at a single private center, and publication costs were covered by the product manufacturer. These factors may introduce bias and limit the generalizability of the findings, highlighting the need for independent, multi-center studies to validate and extend these results.

## 5. Conclusions

This prospective, single-center, unblinded comparison demonstrates that Rennova^®^ Diamond Intense significantly increases dermal thickness, improves skin elasticity, reduces transepidermal water loss, and achieves excellent patient and physician satisfaction. These findings indicate that Rennova^®^ Diamond Intense is an effective, safe, and versatile option for esthetic facial rejuvenation. Practitioners can confidently include it in their range of fillers to meet patient needs, preferences, and practical considerations without compromising clinical outcomes. Future studies with larger sample sizes and longer follow-up are warranted to further investigate its long-term biostimulatory effects and reinforce the current evidence base.

## Figures and Tables

**Table 1:** Summary of inclusion and exclusion criteria.

Inclusion Criteria	Exclusion Criteria
▴ Adults aged 30 to 75 years	
▴ Visible signs of mild to moderate facial aging (e.g., volume loss, skin laxity, rhytids)	▴ Pregnant or breastfeeding women
▴ Fitzpatrick skin types I to IV	▴ History of autoimmune disease
▴ No known hypersensitivity to filler components or local anesthetics	▴ Use of immunosuppressive or anti-inflammatory medications
▴ Willingness and ability to comply with follow-up and study procedures	▴ Presence of permanent cortical implants (e.g., PMMA or silicone)
▴ Provided written informed consent	▴ Temporary or semi-permanent fillers received within the past 12 months
▴ Washout period of ≥12 months for prior esthetic facial treatments or procedures	▴ Use of topical or oral agents affecting skin condition
▴ No additional esthetic facial treatments during the study period	

**Table 2:** Full content of the Global Aesthetic Improvement Scales used.

Score	GAIS Descriptor	Definition
1	Exceptional Improvement	Optimal cosmetic result, significant esthetic enhancement
2	Very Improved	Marked esthetic improvement, but not optimal
3	Improved	Noticeable improvement, but still room for further enhancement
4	No Change	No visible difference compared to pre-treatment
5	Worse	Appearance declined relative to pre-treatment

## Data Availability

Data used in this study are available from the primary author upon reasonable request.

## Abbreviations

The following abbreviations are used in this manuscript:

ANOVA	Analysis of Variance
CAAE	Certificado de Apresentação de Apreciação Ética (Ethics Presentation Certificate)
CaHA	Calcium Hydroxylapatite
IQR	Interquartile Range
P-GAIS	Physician Global Aesthetic Improvement Scale
PMMA	Polymethyl Methacrylate
S-GAIS	Subjective Global Aesthetic Improvement Scale
SPSS	Statistical Package for the Social Sciences
TEWL	Transepidermal Water Loss
